# Applying time series modeling to assess the dynamics and forecast monthly reports of abuse, neglect and/or exploitation involving a vulnerable adult

**DOI:** 10.1186/s13690-020-00431-0

**Published:** 2020-06-08

**Authors:** Nelís Soto-Ramírez, Janet Odeku, Courtney Foxe, Cynthia Flynn, Diana Tester

**Affiliations:** 1 The Center for Child and Family Studies, College of Social Work, University of South Carolina, 226 Bull St, Columbia, SC 29208 USA; 2 Department of Social Services, P.O. Box 1520, 1535 Confederate Ave, Columbia, SC 29202 USA

**Keywords:** Forecast, Modeling, Auto-regressive integrated moving average (ARIMA), Vulnerable adults

## Abstract

**Background:**

Application of time series modeling to predict reports related to maltreatment of vulnerable adults can be helpful for efficient early planning and resource allocation to handle a high volume of investigations. The goal of this study is to apply: (1) autoregressive integrated moving average (ARIMA) time series modeling to fit and forecast monthly maltreatment reports accepted for assessment reported to adult protective services (APS), and (2) interrupted time series analysis to test whether the implementation of intake hubs have a significant impact in the number of maltreatment reports after the implementation period.

**Methods:**

A time series analysis on monthly APS intake reports was conducted using administrative data from SC Child and Adult Protective Services between January 2014 and June 2018. Monthly APS data were subjected to ARIMA modeling adjusting for the time period when intake hubs were implemented. The coefficient of determination, normalized SBC, AIC, MSE, and Ljung-Box Q-test were used to evaluate the goodness-of-fit of constructed models. The most parsimonious model was selected to predict the monthly APS intakes from July to December 2018. Poisson regression was fit to examine the association of the implementation of the hubs and the number of intake reports received to APS, adjusting for confounders.

**Results:**

Over 24,000 APS intakes accepted for investigation were identified over a period of four calendar years with an increase in the monthly average of APS intakes between 2014 and 2017. An increase in the number of monthly APS intakes was found after the intake hubs were implemented in 2015 (Phase-1) and 2017 (Phase-2). Of all the models tested, an ARIMA (12), 1, 1 model was found to work best after evaluating all fit measures for both models. For Phase-1, the optimum model predicted an average of 488 APS intake reports between July and December 2018, representing a 9% increase from January–June 2018 (median = 445). For Phase-2, the percent increase was 32%.

**Conclusions:**

The implementation of the intake hubs has a significant impact in the number of reports received after the implementation period. ARIMA time series is a valuable tool to predict future reports of maltreatment of vulnerable adults, which could be used to allow appropriate planning and resource allocation to handle a high volume of monthly intake reports.

## Background

Time series models have been traditionally used as a forecasting technique in various disciplines such as public health and economics [1–5]. For instance, Zhang and colleagues evaluated and compared the performances of artificial neural network models and auto-regressive integrated moving average (ARIMA) models for modeling and predicting future epidemic events [3]. One of the advantages of using ARIMA models over simple regression models is that the former takes into consideration the periodic variations, underlying changing trends and random disturbances of a time series, and use the associations in the sequentially lagged relationships to predict future values [2, 6, 7]. Forecasting techniques used in epidemiological studies could be also applied in social welfare research. Application of time series modeling to predict reports related to maltreatment of vulnerable adults can be beneficial for efficient early planning and resource allocation to handle high volume of investigations.

For the purpose of this study, intake data from the South Carolina (SC) Child and Adult Protective Services System (CAPSS) administrative database was used to apply ARIMA time series analysis to fit and forecast monthly intake reports related to maltreatment of vulnerable adults reported to Adult Protective Service (APS). Prior to 2015, the Department of Social Services (DSS) received calls for investigation of possible adult and child abuse and/or neglect through its county offices (46 counties). To improve the consistency of intake decisions across the state, DSS began planning for and operationalizing a streamlined process of all intake calls to be managed and evaluated in the five regional intake hubs. This multi-year project had two phases. In the first phase, from January 2015 to January 2016, DSS rolled 22 counties into the regional hubs, and in the second phase, the remainder of the counties (*n* = 24) were implemented from May 2017 to November 2017. As a result, an increase in APS intake reports was observed after 2015. A second wave or spike was detected in the first few months of 2018. Hence, for the time series analysis we separated the analysis into two time series, one for the 22 counties that became centralized into intake hubs in 2015 (Phase 1), and the other for the 24 counties that were centralized into intake hubs in 2017 (Phase 2). These two time series were used to assess the impact of the intake hubs and to predict the expected monthly APS reports from July 2018 to December 2018. Hence, in this study we aim: (1) to apply interrupted time series analysis to test whether the implementation of intake hubs have a significant impact in the number of maltreatment reports after the two implementation periods (Phase 1 & 2), and (2) then to apply ARIMA time series modeling to fit and forecast (predict) monthly maltreatment reports accepted for assessment reported to APS in South Carolina.

## Methods

### Study design and data source

This is a retrospective study of all APS intakes accepted for assessment between January 1st 2014 and June 30th 2018 in South Carolina, USA. Monthly APS intakes is the principal outcome variable of the study. Data were obtained retrospectively from administrative files of the South Carolina CAPSS system.

### APS criteria of a vulnerable adult

A ‘vulnerable adult” is a person 18 years of age or older who has a physical or mental condition which prevents them from providing for his or her own care or protection. This include adults who are impaired because of brain damage, advanced age, and physical, mental or emotional dysfunction [8].

### Criteria to accept a report or referral for investigation

For a report or referral to meet the criteria to be accepted for investigation, there must be an allegation that a vulnerable adult is being maltreated through abuse, neglect, self-neglect, or exploitation, in a community setting. The APS Intake Tool must be used to determine if the report meets the legal criteria for vulnerability.

### Data analysis

Descriptive statistics included frequency distributions, means, medians, and 95% confidence intervals (CIs) of APS intakes. For the time series analysis, Box-Jenkins (1970) [9] approach was used to fit the best ARIMA (*p, d, q*) model for monthly APS intakes accepted for assessment from January 2014 to June 2018. The data from January 2014 to December 2017 (in-sample data) were used for training the forecasting model, and the validation was performed on the monthly APS intakes from January 2018 to June 2018 (out-of-sample data). To re-estimate all parameters of the selected ARIMA model, all data from January 2014 to June 2018 was used to forecast intake reports for the subsequent 6 months.

ARMA models are a combination of auto-regression (AR) and moving average (MA) models, in which the current value of the time series is expressed linearly in terms of its previous values as well as current and previous residual series [10]. The ARIMA model deals with non-stationary time series with differencing process based on the ARMA model. This model-building process is designed to make use of previous observations to make predictions of future values using lag parameter values, under the assumption that the pattern will persist.

The parameter for the model includes *p,* the order of AR; *d,* the order of difference (integration) and *q,* the order of MA. To obtain the best model, three steps were followed: model specification (identification), model fitting (estimation), and model diagnostics [11]. The *identification* stage describes the serial correlation of the data and any relations to external factors. Lagged scatter-plots were evaluated and the augmented Dickey-Fuller (ADF) unit root test was used to identify whether or not the time series was stationary. If the ADF test indicates that the series is non-stationary, it is required to take differences and continue the model-building process with the differenced series. Also, to stabilize the variance, the monthly counts of APS intakes were transformed using a quartic root transformation.

Once a stationary series was obtained, the AR and MA orders were determined after the examination of the autocorrelation function (ACF) and the partial autocorrelation function (PACF) plots. The ACF measures whether earlier values in the series have some relation to later values. PACF captures the amount of correlation between a variable and a lag of itself that is not explained by correlations at all lower order lags [3]. Once a set of candidate models were identified, we proceeded with parameter estimation and model diagnostics. To compare different ARIMA models the following measures of overall fit were evaluated: (1) coefficient of determination (R^2^), (2) Akaike Information Criterion (AIC), (3) Schwartz Bayesian Criterion (SBC), and (4) Mean Square Error (MSE). The best model is the one with the highest R^2^ and the lowest AIC, SBC, and MSE. In the *model fitting* step, we estimated the parameters of the model selected and then tested for significance of the parameter estimates. The parameters were estimated with the maximum likelihood (ML) method after the identification step [12].

In the *model diagnostic* step, the goodness of fit was examined by means of Ljung-Box Q-test and by plotting the ACF of the residuals of the fitted model. If the model is adequate then residuals should be uncorrelated (white-noise) and Q should be small. A non-significant value indicates that the chosen model fits well. If more than 5% of the autocorrelation fall outside this range then the residual process is not white noise.

#### Exogenous variables

Exogenous variables were added to the ARIMA model to assess the impact of the implementation of the intake hubs. This exogenous variable is an event that may influence the number of APS intakes reported after 2015 and 2017. Hence, this event may have a temporary effect on the number of intake reports after 2015 and 2017 or a more permanent effect. Coefficients and standard errors of the exogenous variables will show whether the effect of the intake hubs on monthly APS intake reports is significant, while the AIC and SBC will indicate whether the model improves compared to the univariate model [10]. The residuals of the models should mimic white noise.

#### Forecasting future reports

To predict the future values, the most parsimonious developed ARIMA model was fitted to the entire data from January 2014 to June 2018 and used to forecast over a time span of 6 months, covering July 2018 and December 2018. In the *forecasting* stage, we predicted subsequent observations and their corresponding prediction interval (95% prediction interval) for both time series. In terms of *y*, the general forecasting equation [13] is:

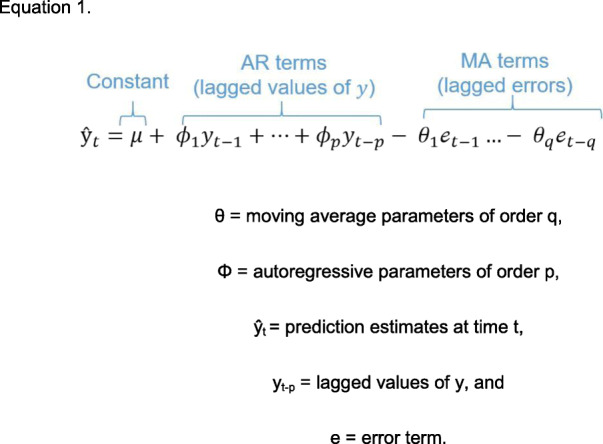



To obtain the predicted values in the original scales, the reverse transformation was calculated. Data were analyzed using SAS Software Version 9.4 (North Carolina State University, Raleigh, NC, USA) and the level of significance was set at 5%.

### Poisson regression

Poisson regression models with a robust error variance were fit to examine the association of the implementation of the hubs in 2015 and 2017 and the number of intakes reports received to APS, adjusting for gender (proportion of males), race (proportion of being Black), and age (median age) of the clients. The SAS PROC GENMOD procedure with REPEATED statement was used to obtain robust standard errors for the poisson regression coefficients. Least squares means were estimated to get the predicted number of intakes (predicted counts).

## Results

**Fig. 1:**
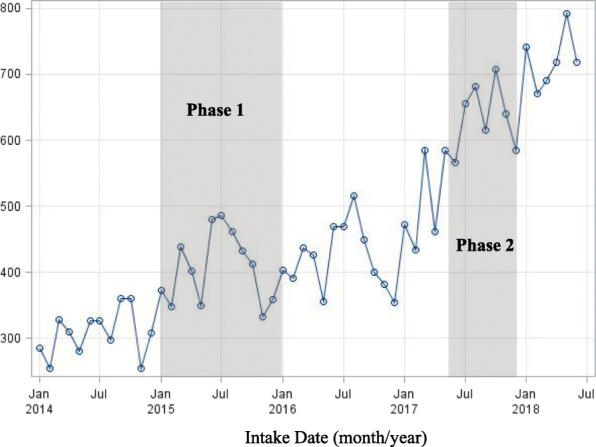
Trend of monthly APS intakes accepted for assessment between January 2014 and June 2018. Gray bars indicate the time period when the Hubs were implemented

**Fig. 2:**
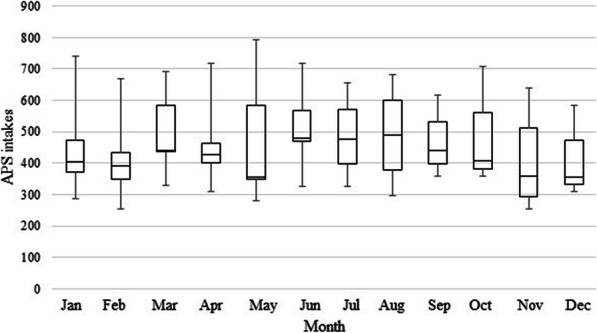
Box-plot distribution of median monthly APS intakes accepted for assessment. Line within the box represents median values, border lines represent the first and the third quartile

In 2015, all APS intakes for 22 of 46 counties became centralized into intake hubs. As a result, an increase of 32.2% in monthly APS intakes was observed in 2015. The remaining 24 counties were rolled into intake hubs in May 2017. An increase of 38.4% in monthly APS intakes was evident in 2017 (Table 1 and Fig. 1). Figure 2 depicts the median of monthly APS intake reports from 2015 to 2018. It is noticeable that the highest average of monthly APS intake reports was registered from June to August (Median: June - 480, July – 478, August – 489), followed by March (Median: 438).

**Table 1:** Distribution of APS intakes accepted for assessment with Intake Hubs implemented in 2015 and 2017

			Intake Hubs implemented from January 2015 to January 2016 Phase 1 (22 counties)				Intake Hubs implemented from May 2017 to November 2017 Phase 2 (24 counties)			
Calendar Year	# of Intakes State	Pct. Increase	# of Intakes	Median	Percentile 25th,75th	Quartile Range	# of Intakes	Median	Percentile 25th,75th	Quartile Range
2014	3676	–	2375	191	183, 221	38	1301	106	93, 124	32
2015	4861	32.2	3574	312	256, 335	78	1287	109	95, 119	24
2016	5052	3.9	3580	304	271, 315	44	1472	122	104, 136	32
2017	6990	38.4	4728	401	349, 428	80	2262	188	153, 225	72
2018 (Jan-Jun)	4331	–	2665	446	422, 462	40	1666	272	264, 294	30

**Fig. 3:**
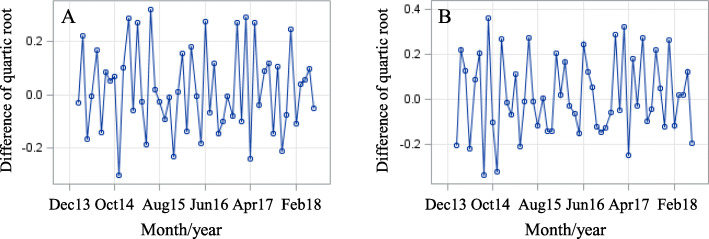
Stationary Phase 1 & 2 time series; first differences of quartic root transformed data. **a**: Phase 1; January 2015 to January 2016–22 counties. **b**: Phase 2; May 2017 to November 2017–24 counties

The time series plot suggests a non-stationary process with a linear upward trend (Fig. 1). In addition, there is a notable non-constant variance, hence, to stabilize the variance the counts of APS intakes were transformed using a quartic root transformation. The Augmented Dickey-Fuller (ADF) unit root test indicated that the two series are nonstationary (ADF *p*-value ≥0.05), hence the time series were first differentiated. The ACF for both time series decayed very slowly, indicating that it is suitable to take first differences. The first differences of quartic root transformed data $\left\{\nabla \sqrt[4]{Y_t}\right\}$ appears to be stationary (Fig. 3; Panel A (Phase 1) & B (Phase 2)).

### Estimation stage of the ARIMA model

The data was split into training (80%; from January 2014 to December 2017) and testing (20%; from January 2018 to June 2018) sets for both Phase 1 and Phase 2 models. Phase 1 model comprise the 22 counties that became centralized into intake hubs between January 2015 and January 2016, and Phase 2 include data of the 24 counties that were centralized into intake hubs between May 2017 and November 2017. To estimate the ARIMA model we used the testing data (January 2014 to December 2017) and then re-estimated the model parameters using all data (January 2014 to June 2018). Different combinations of AR and MA orders were tested after evaluating the ACFs and PACFs of the stationary series (Table 2). The ACF function decays for the first lag, then it drops off to zero abruptly. Therefore, a MA order of 1 was considered for both time series. Also, an AR order of (1) and (12) were considered based on the PACF plots. Of all the models tested, an ARIMA (12), 1, 1 model (*p* = (12), *d* = 1, *q* = 1) was found to perform best after evaluating all fit measures for both models (Phase 1 model: AIC = − 74.22, SBC = − 68.31, R^2^ = 0.86, MAE = 0.013; Phase 2 model: AIC = − 51.13, SBC = − 45.22, R^2^ = 0.81, MAE = 0.012). The Ljung-Box chi-square statistics and the autocorrelation function of the residuals indicate that the residuals are independent, and the chosen model is appropriate (Phase 1 model P _Box-Ljung (Q)_ = 0.47; Phase 2 model P _Box-Ljung (Q)_ = 0.09).

**Table 2:** ARIMA models and selection criteria

	Phase 1: January 2015 to January 2016 (22 counties)					Phase 2: May 2017 to November 2017 (24 counties)				
ARIMA Model	AIC	SBC	R^**2**^	MSE	Ljung_Box ***p***-value	AIC	SBC	R^**2**^	MSE	Ljung_Box ***p***-value
**p(12); q = 1;** **d = 1**	**−74.22**	**−68.31**	**0.86**	**0.013**	**0.47**	**−51.13**	**−45.22**	**0.81**	**0.020**	**0.09**
p = 0; q (1) (12); d = 1	- 70.94	−65.03	0.85	0.014	0.42	−49.52	−43.61	0.81	0.020	0.05
p (1) (12); q = 0; d = 1	−74.61	−68.70	0.86	0.013	0.16	−44.19	−38.28	0.78	0.023	0.004

The constructed ARIMA models include a positive AR component (Phase 1 coefficient = 0.561, *p* < 0.0001; Phase 2 coefficient = 0.343, *p* = 0.016) indicating that current month’s number of APS intakes depends on the average value of APS intakes plus some fraction of its deviation from this average value a year ago, plus a random error (Table 3). In addition, a positive MA component with a lag of one (Phase 1 coefficient = 0.552, *p* < 0.0001; Phase 2 coefficient = 0.695, p < 0.0001) indicates that each value of the variable is determined by the current disturbance and previous forecast errors.

**Table 3:** Parameter estimates of the selected ARIMA model for APS intakes (# of observations = 53)

	Phase 1: January 2015 to January 2016 (22 counties)			Phase 2: May 2017 to November 2017 (24 counties)			
Parameter	Estimate	Standard Error	***P***-value	Estimate	Standard Error	***P***-value	Lag
MA1,1	0.552	0.115	< 0.0001	0.695	0.116	< 0.0001	1
AR1,1	0.561	0.117	< 0.0001	0.343	0.142	0.016	12
Hub	0.279	0.089	0.001	0.043	0.015	0.004	0

The ARIMA models improve after adding the exogenous variables that takes the value ‘0’ before the hub implementation time period and ‘1’ after that. The impact of the hub implementation variables are significant (Phase 1 coefficient = 0.279, *p* = 0.001; Phase 2 coefficient = 0.043, *p* = 0.004), showing a significant increase on monthly APS reports after the implementation periods (Table 3). Clearly, the dynamics of APS intake reports was influenced by the implementation of the intake hubs. This model was used for forecasting APS intake reports into the future.

### Forecasting stage

**Fig. 4:**
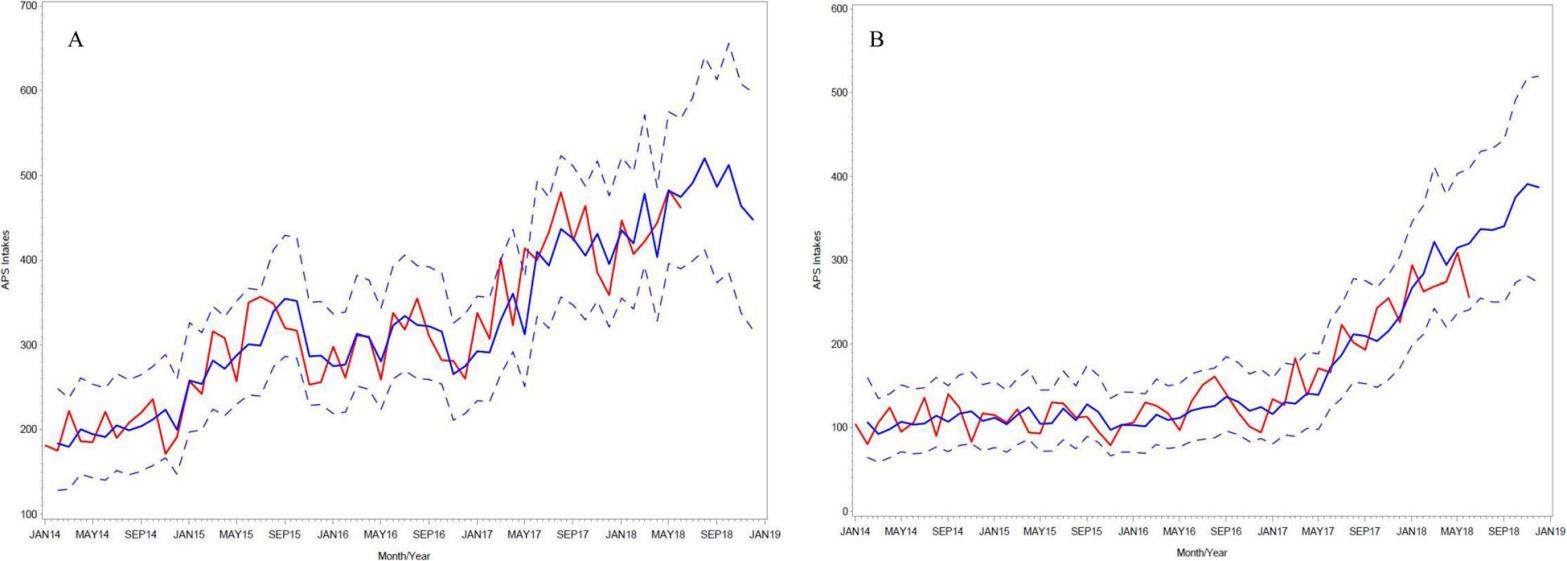
Monthly time series for observed and fitted APS intakes, and forecast intakes for July–December 2018. **a**: Phase 1: January 2015 to January 2016–22 counties. **b**: Phase 2: May 2017 to November 2017–24 counties. Red = actual. Blue = forecast. Blue-dash = 95% prediction interval

The training data (January 2014 to December 2017) successfully predicted the monthly APS intake reports between January 2018 and June 2018 (testing set; “out-of-sample forecast”), in which the testing set fell within the 95% confidence interval (see Additional file 1; Table 1). After determining the optimal model, the monthly forecast of APS intake reports and the 95% prediction intervals were calculated for the time period of July 2018 to December 2018 (Table 4). For Phase 1, the optimum ARIMA (12), 1, 1 model predicted an average of 488 APS intake reports (95% prediction interval between 447 and 520) between July and December 2018, representing a 9% increase from January–June 2018 (median = 445). For Phase 2, the percent increase from January–June 2018 (median = 272) to July–December 2018 (median = 358; 95% prediction interval: 336, 391) was 32%. Fig. 4 displays the actual number of APS intakes and the prediction from the ARIMA model with the corresponding 95% predicted intervals (Panel A: Phase 1 & Panel B: Phase 2 models). Major peaks can be observed around June to August, and again a light peak for March, adequately capturing the pattern in the data.

**Table 4:** Forecasted monthly APS intakes accepted for assessments

Lead times	Phase 1: January 2015 to January 2016 (22 counties)		Phase 2: May 2017 to November 2017 (24 counties)	
**Date**	**Predicted APS intakes**	**95% CI**	**Predicted APS intakes**	**95% CI**
July 2018	491	398, 592	337	254, 430
August 2018	520	412, 640	336	250, 433
September 2018	486	373, 613	340	250, 444
October 2018	512	385, 656	375	273, 491
November 2018	464	338, 608	391	280, 517
December 2018	447	318, 596	387	272, 519
	**Median**	**95% CI**	**Median**	**95% CI**
July – December 2018	488	447, 520	358	336, 391
January – June 2018	446	407, 483	272	256, 309
Percent increase	9%	–	32%	–

### Poisson regression

To corroborate the significant effect of the implementation of the hubs on the increase of cases reported to APS found in the ARIMA model, poisson regression analysis was performed after adjusting for gender (proportion of males), race (proportion of being Black), and age (median age) of the clients. The least square means of the estimates are shown in Table 2 (see Additional file 1; Table 2). It seems that the predicted number of intakes after the implementation of the hubs in 2015 was (203.6/143.58) = 1.41 times the predicted number of intakes before the implementation of the hubs in 2015. Likewise, the predicted number of intakes after the implementation of the hubs in 2017 was (107.33/64.24) = 1.67 times the predicted number of intakes before the implementation of the hubs in 2017. These results validate the significant effect of the implementation of the hubs on the number of intakes reported to APS that were found in the ARIMA models.

## Discussion

With ARIMA modeling, we successfully assessed the dynamics and fluctuations of the APS intake reports and accurately forecasted an increase in reports of maltreatment of vulnerable adults into the future. We found that the implementation of the intake hubs have a significant impact in the number of reports received after the implementation period. This exogenous variable (hub implementation) added explanatory power to the ARIMA process, substantially improving the ARIMA model.

We found that ARIMA (12), 1, 1 model is the most appropriate model to predict APS intake reports into the future. The results indicate that the predicted data by ARIMA model are quite similar to the actual data during early 2018 calendar year. Our findings also revealed that monthly APS intakes are more frequent during March and over the summer (June to August). It seems that the implementation of intake hubs in 2015 and 2017 boosted the number of APS intake reports onward. The ARIMA predictive model showed an ascending trend of these reports, indicating a necessity for appropriate planning, allocation of funding and investigators to handle a large volume of APS intakes in the future. Not only a high demand of social workers are needed, but an increase in health care expenditures among this population is expected.

Based on projections made by the South Carolina Revenue and Fiscal Affairs, South Carolina is expected to reach aging population status by the year 2030, at which point 22% of the total state’s population will be 65 years or older [14]. As the number of older adults is projected to increase dramatically over the next 10 years, this will pose major challenges to our social service agency, as there could be greater reports of maltreatment of vulnerable adults among this population making this a social and public health issue. In our study, around 63% of the cases were 65 years of age or older, in which 62% were females. This implies a higher need of social workers specialized in vulnerable older adults and gerontology.

One limitation of this study is that the univariate forecasting ARIMA model was used. However, the significant effect of the implementation of the hubs on the number of cases reported to APS were corroborated with the poisson regression models after adjusting for age, gender, and race. Nevertheless, the goal of this paper is to evaluate the dynamics and fluctuations of APS intake reports over time and the impact of the implementation of the intake hubs on future intake reports of vulnerable adults. While multiple statistical modeling methods exist for analyzing caseload data [15], we selected time series modeling and forecasting as an appropriate method to show the trend of APS intake reports over a 4-year period.

## Conclusions

In summary, ARIMA time series modeling is a valuable tool for forecasting future reports of maltreatment of vulnerable adults with a high accuracy. Policymakers and program administrators at both the state and federal levels need effective forecasts of future intake reports which could help improve their ability to respond efficiently to high volume of maltreatment reports as South Carolina aging population continues to increase. Forecasting methods can be integrated into routine surveillance practice in social service agencies. More research on the accurate prediction of future intake reports of maltreatment should be conducted and compared with other forecasting techniques.

## Supplementary information

**Additional file 1 **: **Table S1**. Forecasted monthly APS intakes accepted for assessments; training and testing samples.

**Additional file 2 **: **Table S2**. Adjusted predicted least squares means of the number of intakes after the implementation of the hubs, a poisson regression model.

## Data Availability

The datasets used and/or analysed during the current study are available from the corresponding author on reasonable request.

## Abbreviations

ARIMA: Autoregressive integrated moving average
APS: Adult protective services
DSS: Department of Social Services
AR: Auto-regression
MA: Moving average
ML: Maximum likelihood
ACF: Autocorrelation function
PACF: Partial autocorrelation function
ADF: Augmented Dickey-Fuller
R^2^: Coefficient of determination
AIC: Akaike Information Criterion
SBC: Schwartz Bayesian Criterion
MSE: Mean Square Error
