# Supplementary material for: Applying time series modeling to assess the dynamics and forecast monthly reports of abuse, neglect and/or exploitation involving a vulnerable adult
Source: Arch Public Health. 2020 Jun 8;78:53. doi: 10.1186/s13690-020-00431-0 (PMC7278192; doi:10.1186/s13690-020-00431-0)
Supplement: Supplementary file 1 — Additional file 1 : Table S1. Forecasted monthly APS intakes accepted for assessments; training and testing samples. [file 13690_2020_431_MOESM1_ESM.docx]

**Table 1.** Forecasted monthly APS intakes accepted for assessments; training and testing samples

|  | **Phase 1: January 2015 to January 2016**  **(22 counties)** | | | **Phase 2: May 2017 to November 2017**  **(24 counties)** | | |
| --- | --- | --- | --- | --- | --- | --- |
| **Date** | **Actual cases** | **Predicted APS intakes** | **95% CI** | **Actual cases** | **Predicted APS intakes** | **95% CI** |
| January 2018 | 447 | 438.1 | 351.6, 533.2 | 294 | 233.7 | 167.1, 311.4 |
| February 2018 | 407 | 415.8 | 321.8, 520.8 | 263 | 230.4 | 160.4, 312.9 |
| March 2018 | 422 | 485.7 | 368.7, 616.7 | 269 | 258.4 | 177.6, 353.9 |
| April 2018 | 444 | 429.5 | 314.4, 560.7 | 274 | 237.6 | 158.3, 332.8 |
| May 2018 | 482 | 495.9 | 358.1, 653.1 | 309 | 228.6 | 148.3, 326.1 |
| June 2018 | 462 | 487.0 | 342.9, 653.1 | 256 | 226.8 | 143.9, 328.4 |
