# Supplementary material for: Applying time series modeling to assess the dynamics and forecast monthly reports of abuse, neglect and/or exploitation involving a vulnerable adult
Source: Arch Public Health. 2020 Jun 8;78:53. doi: 10.1186/s13690-020-00431-0 (PMC7278192; doi:10.1186/s13690-020-00431-0)
Supplement: Supplementary file 2 — Additional file 2 : Table S2. Adjusted predicted least squares means of the number of intakes after the implementation of the hubs, a poisson regression model. [file 13690_2020_431_MOESM2_ESM.docx]

**Table 2.** Adjusted predicted least squares means of the number of intakes after the implementation of the hubs, a poisson regression model

|  | **Phase 1: January 2015 to January 2016 (22 counties)** | | | **Phase 2: May 2017 to November 2017**  **(24 counties)** | | |
| --- | --- | --- | --- | --- | --- | --- |
| **Variable** | **Mean** | **Standard Error of Mean** | **P-value** | **Mean** | **Standard Error of Mean** | **P-value** |
| After implementation | 203.6 | 5.33 | <0.0001 | 107.33 | 7.780 | <0.0001 |
| Before implementation | 143.58 | 8.47 | <0.0001 | 64.24 | 2.111 | 0.016 |

Adjusted for the proportion of males, proportion of race = black, and the median age of the client reported to APS by month and year.
